# Clinico-microbiological assessment of necrotizing otitis externa in subjects with diabetes mellitus in India

**DOI:** 10.6026/973206300222143

**Published:** 2026-04-30

**Authors:** Rajeev Kumar Nishad, Reetu Verma, Priyanka Das

**Affiliations:** 1 Department of ENT, Autonomous State Medical College, Firozabad, Uttar Pradesh, India; 2 Department of Anaesthesiology, Autonomous State Medical College, Firozabad, Uttar Pradesh, India; 3 Department of ENT, Himalaya Medical College and Hospital, Patna, Bihar, India

**Keywords:** Diabetes mellitus (DM), otitis externa (OE), necrotizing otitis externa (NOE), *Pseudomonas*, pus

## Abstract

Necrotizing otitis externa (NOE) represents a severe progression of otitis externa, particularly threatening in diabetic patients, with limited clinico-microbiological data for Indians. Therefore, it is of interest to evaluate the clinico-microbiological profile in 60 diabetic adults diagnosed with NOE at a tertiary center. Among participants (mean age 60.51±9.6 years; 60% male), universal findings included external auditory canal edema, otorrhea and severe nocturnal otalgia; pus cultures grew *Pseudomonas spp.* in 43.3% (n=26), with no growth in 13.3% (n=8), mild conductive hearing loss in 16.7% (n=10) and facial nerve palsy (with/without glossopharyngeal involvement) in 6.7% (n=4). *Pseudomonas* dominance and characteristic symptoms align with global patterns, but high complication rates underscore local management gaps. Thus, we report the Indian cohort profile of NOE in diabetes, informing targeted antibiotic strategies and early cranial nerve monitoring.

## Background:

OE (otitis externa), sometimes referred to as tropical ear and also known as swimmer's ear, is an infection and inflammation of the external ear canal. Necrotizing otitis externa (NOE) is a severe form of otitis externa that may initially present with only mild inflammation. NOE results from disruption of the cerumen layer or the protective epidermis of the external ear canal, which occurs more frequently in settings with warmer temperatures and increased humidity. Three distinct clinical stages of otitis externa have been described: pre inflammatory, acute inflammatory and chronic [1]. The pre inflammatory stage follows local trauma or moisture exposure in the ear canal. At this stage, the glands are destroyed and the skin becomes edematous, rendering the ear more vulnerable to additional trauma. The acute inflammatory stage of otitis externa is subdivided into mild, moderate and severe forms. In the mild acute inflammatory stage, the canal shows erythema and mild edema, often with pruritus and minimal discomfort. In the moderate acute inflammatory stage, increased discomfort, edema and seropurulent secretions are present. Severe inflammatory otitis externa (OE) is characterized by marked pain; a canal lumen filled with debris and draining secretions and is typically associated with periauricular edema and regional adenopathy. Necrotizing otitis externa refers to extension of the infection to adjacent soft tissues and bone [2]. Chronic otitis externa is defined as a single episode lasting more than 4 weeks or at least 4 episodes within 1 year. Diabetes mellitus is considered an important risk factor for various bacterial infections [3]. Necrotizing otitis externa is a life threatening skull base infection that involves the external ear canal and surrounding soft tissues. Temporal bone osteomyelitis associated with necrotizing otitis externa was first described in 1838. Reports in the existing literature describe cases of acute skull base osteomyelitis in individuals with diabetes presenting with purulent otorrhea and auricular necrosis [4]. The causative organism *Bacillus pyocyaneus*, now known as *Pseudomonas* aeruginosa, was first identified in association with this condition and the term malignant was subsequently introduced to reflect its poor prognosis. However, some studies have preferred the terms necrotizing or invasive otitis externa to emphasize that necrotizing otitis externa is not a neoplasm. Fungal infections of the external ear, involving the auricle, external auditory canal, tympanic membrane and sometimes the middle ear, are collectively referred to as otomycosis. An infection of the external auditory canal that extends into the mastoid air cells and skull base is termed invasive necrotizing otitis externa [5]. Therefore, it is of interest to evaluate the clinic-microbiological profile of individuals with diabetes mellitus (DM) who had necrotizing otitis media.

## Materials and Methods:

The goal of this prospective observational clinical examination was to evaluate the clinic-microbiological profile in individuals with diabetes mellitus (DM) who had necrotizing otitis media. The study was conducted at the Department of ENT, F.H. Medical College, Etmadpur, Agra, Uttar Pradesh. Before participation, each subject provided both written and verbal informed consent. The study assessed all subjects diagnosed with otitis externa and diabetes mellitus who presented to the Institute within the defined study period. Otitis externa's diagnosis was made using the clinical findings and necrotizing otitis externa diagnosis was made with both radiographic and clinical findings based on obligatory/major and occasional/minor diagnostic criteria. A diagnosis of necrotizing otitis externa was made when all major criteria were met. The data assessed in the study subjects included gender, comorbidities, age and involvement of the cranial nerves, using the Brackman score to grade the facial nerve. The study also assessed critical laboratory test values, including ESR (erythrocyte sedimentation rate), CRP (C-reactive protein), WBC (white blood cell) count, HbA1c (glycated hemoglobin) levels and blood glucose levels. The imaging techniques used include HRCT (high-resolution computed tomography) when indicated. Subjects with diabetes were assessed in the study based on their medical history. In all subjects, the external auditory canal was cleaned and examined microscopically. In all the subjects, cultures were collected. In all subjects, topical and systemic antibiotic therapy was given, with modification based on swab culture and histological findings. In all the subjects, oral antibiotic therapy was given for a minimum of 6 weeks after discharge from the hospital. Using SPSS (Statistical Package for the Social Sciences) software version 24.0 (IBM Corp., Armonk, NY, USA), the collected data were statistically evaluated using descriptive statistics, the Student's t-test, ANOVA, the Mann-Whitney U test, the Chi-square test and the Pearson correlation coefficient. The percentage, frequency, mean and standard deviation were used to express the results. A p-value of less than 0.05 was taken into account.

## Results:

In this study, sixty individuals with a confirmed diagnosis of diabetes mellitus and otitis externa were evaluated. Males were 60% (n=36) and females were 40% (n=24). No study subject was in the age range of 21-30 and 31-40 years, 20% (n=12), 26.66% (n=16), 33.33% (n=20) and 0 subjects in 41-50, 51-60, 61-70, 71-80 and 81-90 years of age, respectively (Table 1 - see PDF). In responders, no growth was observed in 13.33% of study subjects (n=8) on the pus culture. The highest number of organisms was seen as *Pseudomonas* species in 43.33% (n=26) study subjects, followed by Staphylococcus species in 16.66% (n=10) study subjects, Streptococcus species and *Candida* species in 10% (n=6) study subjects and *Klebsiella* species in 6.66% (n=4) study subjects, respectively (Table 2 - see PDF). The study results showed that for PTA (pure tone audiometry) in study subjects, mild CHL (conductive hearing loss) was seen in 16.66% (n=10) study subjects and normal hearing on pure tone audiometry was seen in 83.33% (n=50) subjects (Table 3 - see PDF). According to Table 4 (see PDF), the mean blood glucose, HbA1C, CRP, WBC and ESR for the study patients were 174.87 ±40.06, 7.34±1.22, 44.384±18.92, 7545±1001 and 71.064±19.17, respectively.

## Discussion: 

In the current study, there were 40% (n=24) females and 60% (n=36) males. There were no study participants in the 21-30 and 31-40 age groups, 20% (n=12), 26.66% (n=16), 33.33% (n=20) and 0 participants in the 41-50, 51-60, 61-70, 71-80 and 81-90 age groups. This elderly male predominance (60%) aligns with recent cohorts reporting 76-83% males and mean ages of 68-75 years among people with diabetes, confirming near-universal diabetes (95-100%) in Indian NOE [6]. These results were consistent with earlier research by Chen *et al.* (2014) [7] and Kaya *et al.* (2018) [8], in which the authors considered participants with diabetes mellitus and otitis externa with similar demographic characteristics. Contemporary series reinforce this pattern, emphasizing the rising burden in uncontrolled diabetics [9]. The study results showed that, for pus culture, no growth was observed in 13.33% of study subjects (n=8). The highest number of organisms was observed in *Pseudomonas* species, with 43.33% (n=26) of study subjects, followed by Staphylococcus species (16.66% n=10), Streptococcus species and *Candida* species (10% n=6) and *Klebsiella* species (6.66% n=4), respectively. These results were consistent with the findings of Ravikumar *et al.* in (2017) [10] and Grandis *et al.* in (2004) [11], in which the authors' pus culture data matched the present study's results. *Pseudomonas* dominance (43.3%) with secondary *Staph/Streptococcus/Candida* persists amid culture-negatives (13.3%), supporting anti-pseudomonal therapy amid emerging trends [12]. A similar clinico-microbiological study on NOE in diabetic patients by Teronpi *et al.* (2024) reported *Pseudomonas sp.* as the predominant isolate (comparable to our 43.3%), reinforcing the need for empiric anti-pseudomonal therapy in this high-risk group [13]. In the PTA (pure tone audiometry) study, mild CHL (conductive hearing loss) was observed in 16.66% (n=10) of subjects and normal hearing on PTA was observed in 83.33% (n=50) of subjects. These findings align with the results of Rajput *et al.* in (2013) [14] and Shavit *et al.* in (2016) [15], in which PTA (pure tone audiometry) results matching those of the present work were also reported. Mild CHL in 16.7% with normal PTA in 83.3% indicates early disease, consistent with a series where conductive loss predominates pre-skull base involvement [6]. On assessment of the laboratory data in the study subjects, mean blood glucose, HbA1C, CRP (C-reactive protein), WBC (white blood cells) and ESR (erythrocyte sedimentation rate) were 174.87±40.06, 7.34±1.22, 44.384±18.92, 7545±1001 and 71.064±19.17, respectively. These results were consistent with the findings of Yang *et al.* in (2020) [16] and Guerrero-Espejo *et al.* in (2017) [17], where the laboratory data reported in these studies could be compared with the present study results. Moderate HbA1c (7.34±1.22%) and elevated CRP/ESR mirror prognostic markers, where HbA1c ≥8% predicts poor outcomes and serial ESR/CRP monitors response [18]. Recent data highlight increasing NOE with diabetes and shifts in pathogen prevalence. This study advances knowledge by profiling Indian diabetics (*Pseudomonas* 43%, HbA1c 7.34%, mild CHL 17%), advocating glycemic control and monitoring [19].

## Conclusion:

We show that necrotizing otitis externa is common among subjects with diabetes mellitus, particularly those aged 60-80 years. The common species found was among subjects *Pseudomonas*. However, further clinical studies with large subjects are needed.

## References

[1] Korbel L, Spencer JD (2015). J Diabetes Complications..

[2] Ellis J (2024). Can Fam Physician..

[3] Azeez TA, Adeagbo AK (2023). Indian J Otolaryngol Head Neck Surg..

[4] Sylvester MJ (2017). Laryngoscope..

[5] Hutson KH, Watson GJ (2019). J Laryngol Otol..

[6] Costa MB, Onishi ET (2023). Int Arch Otorhinolaryngol..

[7] Chen JC (2014). Scientific World Journal..

[8] Kaya A (2018). Turk Arch Otorhinolaryngol..

[9] Lambor D (2021). Int J Otorhinolaryngol Head Neck Surg..

[10] Ravikumar A (2017). J Med Sci Clin Res..

[11] Grandis JR (2004). Lancet Infect Dis..

[12] https://www.ncbi.nlm.nih.gov/books/NBK556138/.

[13] Teronpi M (2024). Indian J Otology..

[14] https://ecommons.aku.edu/.

[15] Shavit SS (2016). Am J Otolaryngol..

[16] Yang TH (2020). Ann Otol Rhinol Laryngol..

[17] Guerrero-Espejo A (2017). Acta Otorrinolaringol Esp..

[18] Antony S (2021). Int J Otorhinolaryngol Head Neck Surg..

[19] Byun YJ (2020). Otol Neurotol..

